# HIF-Independent Regulation of Thioredoxin Reductase 1 Contributes to the High Levels of Reactive Oxygen Species Induced by Hypoxia

**DOI:** 10.1371/journal.pone.0030470

**Published:** 2012-02-13

**Authors:** Salvador Naranjo-Suarez, Bradley A. Carlson, Petra A. Tsuji, Min-Hyuk Yoo, Vadim N. Gladyshev, Dolph L. Hatfield

**Affiliations:** 1 Molecular Biology of Selenium Section, Laboratory of Cancer Prevention, National Cancer Institute, Bethesda, Maryland, United States of America; 2 Department of Pathology, The Sol Goldman Pancreatic Cancer Research Center, Johns Hopkins Medical Institutions, Baltimore, Maryland, United States of America; 3 Department of Biological Sciences, Towson University, Towson, Maryland, United States of America; 4 Division of Genetics, Department of Medicine, Brigham & Women's Hospital and Harvard Medical School, Boston, Massachusetts, United States of America; University Health Network, Canada

## Abstract

Cellular adaptation to hypoxic conditions mainly involves transcriptional changes in which hypoxia inducible factors (HIFs) play a critical role. Under hypoxic conditions, HIF protein is stabilized due to inhibition of the activity of prolyl hydroxylases (EGLNs). Because the reaction carried out by these enzymes uses oxygen as a co-substrate it is generally accepted that the hypoxic inhibition of EGLNs is due to the reduction in oxygen levels. However, several studies have reported that hypoxic generation of mitochondrial reactive oxygen species (ROS) is required for HIF stabilization. Here, we show that hypoxia downregulates thioredoxin reductase 1 (TR1) mRNA and protein levels. This hypoxic TR1 regulation is HIF independent, as HIF stabilization by EGLNs inhibitors does not affect TR1 expression and HIF deficiency does not block TR1 hypoxic-regulation, and it has an effect on TR1 function, as hypoxic conditions also reduce TR1 activity. We found that, when cultured under hypoxic conditions, TR1 deficient cells showed a larger accumulation of ROS compared to control cells, whereas TR1 over-expression was able to block the hypoxic generation of ROS. Furthermore, the changes in ROS levels observed in TR1 deficient or TR1 over-expressing cells did not affect HIF stabilization or function. These results indicate that hypoxic TR1 down-regulation is important in maintaining high levels of ROS under hypoxic conditions and that HIF stabilization and activity do not require hypoxic generation of ROS.

## Introduction

Hypoxia, defined as a decrease in oxygen levels, is a common feature of physiological and pathological processes, including embryonic development, adaptation to high altitudes, wound healing, inflammation, ischemic diseases and solid cancer growth. In response to hypoxic conditions, mammalian cells undergo adaptive changes through modulation of gene expression in which the hypoxia inducible factors (HIF) play a key role [1]–[2]. HIF is a heterodimeric transcription factor composed of an α subunit, which under normoxic conditions is degraded by the proteasome, and a β subunit, whose protein levels are not affected by hypoxia. The intracellular oxygen level is sensed by a family of prolyl hydroxylases, EGLN1, EGLN2 and EGLN3 (also known as PHD2, PHD1 and PHD3, respectively), which hydroxylate two proline residues at the oxygen-dependent domain (ODD) of HIF-α subunits using oxygen, 2-oxoglutarate and Fe^2+^ as co-substrates [3]–[4]. The hydroxyprolines are then recognized by the Von Hippel Lindau protein (VHL) which promotes the ubiquitination of HIF-α thereby targeting it for proteasome degradation. Under hypoxic conditions, the enzymatic activity of the prolyl hydroxylases is impaired and, therefore, HIF-α is no longer hydroxylated and its interaction and ubiquitination by VHL no longer occurs. This leads to its accumulation, translocation to the nucleus, dimerization with the β subunit and regulation of its target genes.

It is generally accepted that hypoxic conditions modify reactive oxygen species (ROS) levels in cells, although it is still debated in which direction this change occurs. Several studies using different technologies for the measurement of ROS have demonstrated an increase in ROS intracellular levels under hypoxic conditions. The increase of ROS levels upon hypoxia treatment requires a functional complex III of the mitochondrial electron transporter chain (ETC) and hypoxic stabilization of HIF appears to be dependent on the hypoxia-induced generation of ROS [5]–[7]. However, the induction of mitochondrial ROS under hypoxic conditions is controversial. Other studies have shown either a hypoxia-induced decrease in the mitochondrial generation of ROS [8], or no correlation between hypoxic stabilization of HIF and mitochondrial ROS levels [9]–[11].

There are several mechanisms that cells use to maintain the levels of H_2_O_2_ and minimize promiscuous oxidation reactions. Among them, the consumption of H_2_O_2_ by cellular thiols is a major contributor to the thiol redox status of the cell. Two major systems control the thiol redox status in the cell, the thioredoxin system and the glutathione system. H_2_O_2_ is consumed through these two systems by reactions with peroxiredoxins (Prx) and glutathione peroxidase 1 (Gpx1). Several Prxs are dithiol-containing enzymes that are oxidized by H_2_O_2_ to the disulfide, and reduced by thioredoxin, which in turn is reduced by the selenocysteine-containing thioredoxin reductase 1 (TR1). On the other hand, Gpx1 uses selenocysteine to reduce H_2_O_2_, generating a selenenic acid that is reduced using glutathione.

Although ROS can function as second messengers in cell signaling at a low concentration, at high concentrations cells try to alleviate oxidative stress through changes in gene expression [12]. Nrf2 is one of the transcription factors that responds to an increase in ROS levels and, among other antioxidant genes, regulates Gpx2 expression [13]. Therefore, Gpx2 and TR1, among other selenoproteins, play a key role in the maintenance of a redox balance inside the cells. However, little is known about their role in the cellular response to hypoxic conditions.

In this study, we show that hypoxia leads to a significant down-regulation of both, protein and mRNA levels of TR1. Using siRNA to downregulate HIF-1α and iron chelators for inhibiting the activity of prolyl hydroxylases we demonstrate that HIF-1α is not sufficient or necessary for the hypoxia-induced down-regulation of TR1. We further show that TR1 over-expression attenuates the hypoxia-induced increase of ROS, whereas TR1 deficiency potentiates the elevation in ROS levels induced by hypoxia. However, neither TR1 over-expression nor TR1 down-regulation affect HIF stabilization or activity under hypoxic conditions. This study establishes a new mechanism by which high levels of ROS could be maintained under hypoxic conditions and support the notion that hypoxic HIF regulation occurs independently of ROS production.

## Methods

### Cell culture and reagents

DT cells, which were derived from the parental NIH 3T3 cells by expressing the oncogenic k-ras genes [14], were grown in Dulbecco's Minimum Essential Medium (DMEM) (Invitrogen, Carlsbad, CA) supplemented with 10% fetal bovine serum (Gibco Invitrogen) and the mouse breast carcinoma cell line, EMT6, was grown in Waymouth's Medium (Invitrogen) supplemented with 15% fetal bovine serum. In both cell lines, an antibiotic-antimicotic solution (Invitrogen) was added to the culture media and cells were maintained in a humidified atmosphere containing 5% CO_2_ at 37°C. Hypoxia (1% O_2_) was induced by culturing the cells inside a modular incubator chamber (model MIC-101 by Billups-Rothenberg Inc., Del Mar, CA) with inflow and outflow valves that was infused with a mixture of 1% O_2_, 5% CO_2_, and 94% N_2_. Where indicated, Deferoxamine or L-Mimosine (Sigma-Aldrich, St. Louis, MO) was added to the culture media at a concentration of 150 µM and 100 µM, respectively.

### Constructs

TR1 and HIF-1α RNAi target sequences have been reported previously [15], [16]. Sense-antisense primers for HIF-1α and TR1 knockdown were annealed and cloned into the BglII-HindIII sites in pSuper Retro Puro following the manufacturer's instructions (Oligoengine, Seattle,WA). TR1 mRNA was amplified by PCR from pcDNA 3.1 TR1-3mΔ [17] and cloned into SmaI-EcoRI sites of pTriex-4 Hygro Vector. The final TR1 construct with His and S tags was amplified from this vector by PCR with the primers 1+2 (Table 1) and cloned into BamHI-EcoRI sites of the retroviral vector pRV IRES Puro. pRV IRES Puro plamid was generated from the vector pLZR IRES GFP (kindly provided by Dr. Antonio Bernad, CNIC, Madrid, Spain) by substituting the GFP gene for the puromycin resistance gene.

**Table 1:** Oligonucleotide probes used in this study.

No.	Target	Direction	Sequence
1	TR1	F	TTAATGGATCCGGAGATATACCATGGCACACCAT
2	TR1	R	AAATGAATTCTCGTTTTGGACACAAGA
3	HIF1-α	F	ACCTACTATGTCACTTTCCTG
4	HIF1-α	R	TTCTGCTGCCTTGTATGG
5	TR1	F	CAGATGGGGTCTCGGAGGAA
6	TR1	R	GTGACTCTGCACAGATTCCGTCAT
7	VEGF	F	TGCCAAGTGGTCCCAG
8	VEGF	R	GTGAGGTTTGATCCGC
9	ADM	F	GTTTCCGTCGCCCTGATG
10	ADM	R	CCCACTTATTCCACTTCTTTCG
11	Actin	F	CCCAGAGCAAGAGAGG
12	Actin	R	GTCCAGACGCAGGATG

### Retroviral production and cell line infection

GP2-293 cells (Clontech, Palo Alto, CA) were transfected in 60-mm dishes using LipoD293 (SignaGen Laboratories, Gaithersburg, MD) following manufacturer's instructions. 1.5 µg of each retroviral vector and 1 µg of amphotropic envelope expression vector (pVSV-G) were used. Medium was changed 24 h folowing transfection. Cell culture supernatants were harvested after an additional 24 h, filtered with 0.22 µm filters, and diluted (1∶2) with fresh medium. Diluted viral supernatants containing 6 µg/mL polybrene were then added to the cells (plated at a low confluency 24 h prior to the infection) and incubated overnight at 37°C.

### Western blotting

After treatment, cells were washed with ice-cold phosphate-buffered saline (PBS) and harvested with Mammalian Protein Extraction Reagent (M-PER) (Thermo Scientific, Rockford, IL). The amount of protein in cell extracts was measured using the BCA protein assay. Protein lysates were resolved on NuPAGE 4–12% Bis-Tris gels (Invitrogen). Proteins were transferred to nitrocellulose membranes (Invitrogen) using electroblotting procedures. Nitrocellulose membranes were blocked with 5% non-fat dry milk in TBS-T (50 mM Tris, pH 7.6, 150 mM NaCl, 0.1% Tween 20) and incubated overnight at 4°C with the indicated antibodies (anti- HIF-1α, MAB1536 from R&D Systems, Minneapolis, MN; polyclonal anti-TR1; polyclonal anti-actin, sc-1615 from Santa Cruz, Santa Cruz, Ca). Immunolabeling was detected by enhanced chemiluminescence (SuperSignal West Dura, Thermo Scientific) and visualized with a digital luminescent image analyzer (Fujifilm LAS-3000).

### ^75^Se-labeling of cells

Cells were seeded into 6-well plates, incubated for 24 h, labeled with 30 µCi of ^75^Se, cultured under normoxic or hypoxic conditions for 12 h and then harvested and lysed as described above. 30 µg of each sample were applied to a NuPAGE 4–12% Bis-Tris gel, electrophoresed and proteins stained with Coomassie Blue. The gel was then dried and exposed to a PhosphorImager (Molecular Dynamics, GE Healthcare). ^75^Se-labeled selenoproteins were identified by autoradiography.

### Real-time quantitative PCR

Total RNA was extracted from the cells using RNeasy mini Kit (Qiagen, Valencia, CA). cDNA was synthesized from 2 µg of total RNA using Impron II Reverse Transcriptase (Promega, Madison, WI). For real-time quantitative PCR, 1 µl of cDNA was used as template in a 25 µl reaction carried out with the POWER SYBR Master mix kit (Applied Biosystems, Carlsbad, CA) following the manufacturer's instructions. PCR amplifications were carried out in MyiQ Two-Color Real-Time PCR Detection System (BioRad, Hercules, CA). To avoid amplification of potential genomic DNA contaminants, the primer pairs used in this study were chosen to hybridize to different exons of the target genes (Table 1, Primers 3–12). The mRNA levels of target genes were calculated relative to the expression of β-actin.

### Thioredoxin reductase activity

Thioredoxin reductase activity was measured as previously described [18]. Briefly EMT6 or DT cells were grown under normoxic/hypoxic conditions or treated with deferoxamine. After 12 h, cells were harvested and resuspended in phosphate buffer and then sonicated for 30 sec on ice. The TR activity was measured in the extracts by subtracting the time-dependent increase in absorbance at 412 nm. A unit of activity was defined as 1.0 µmol of 5-thio-2-nitrobenzoic acid formed/min/mg protein. Protein concentration was measured using the BCA (Bicinchoninic acid) assay (Thermo Fisher Scientific).

### Intracellular ROS measurement

Cells were seeded into 6-well plates, incubated for 24 h and exposed to 10 µg/ml of 5-(and-6)-chloromethyl-2′7′-dichlorodihydrofluorescein diacetate acetyl ester (H_2_DCFDA) for 30 min. Then the medium was replaced and cells incubated for 12 h under normoxic or hypoxic conditions. H_2_DCFDA-stained cells were harvested with a cell lifter, washed with ice-cold PBS and fluorescence of DCFDA measured by flow cytometry using a FACSCalibur 2 cytometer (Becton Dickinson, Franklin Lakes, NJ).

### Statistical analyses

Data are presented as the mean ± SE. Differences between means were determined by Student's t-test using Microsoft Excel. Differences were considered statistically significant at p<0.05.

## Results

### Hypoxia down-regulates TR1 mRNA and protein levels

**Figure 1:**
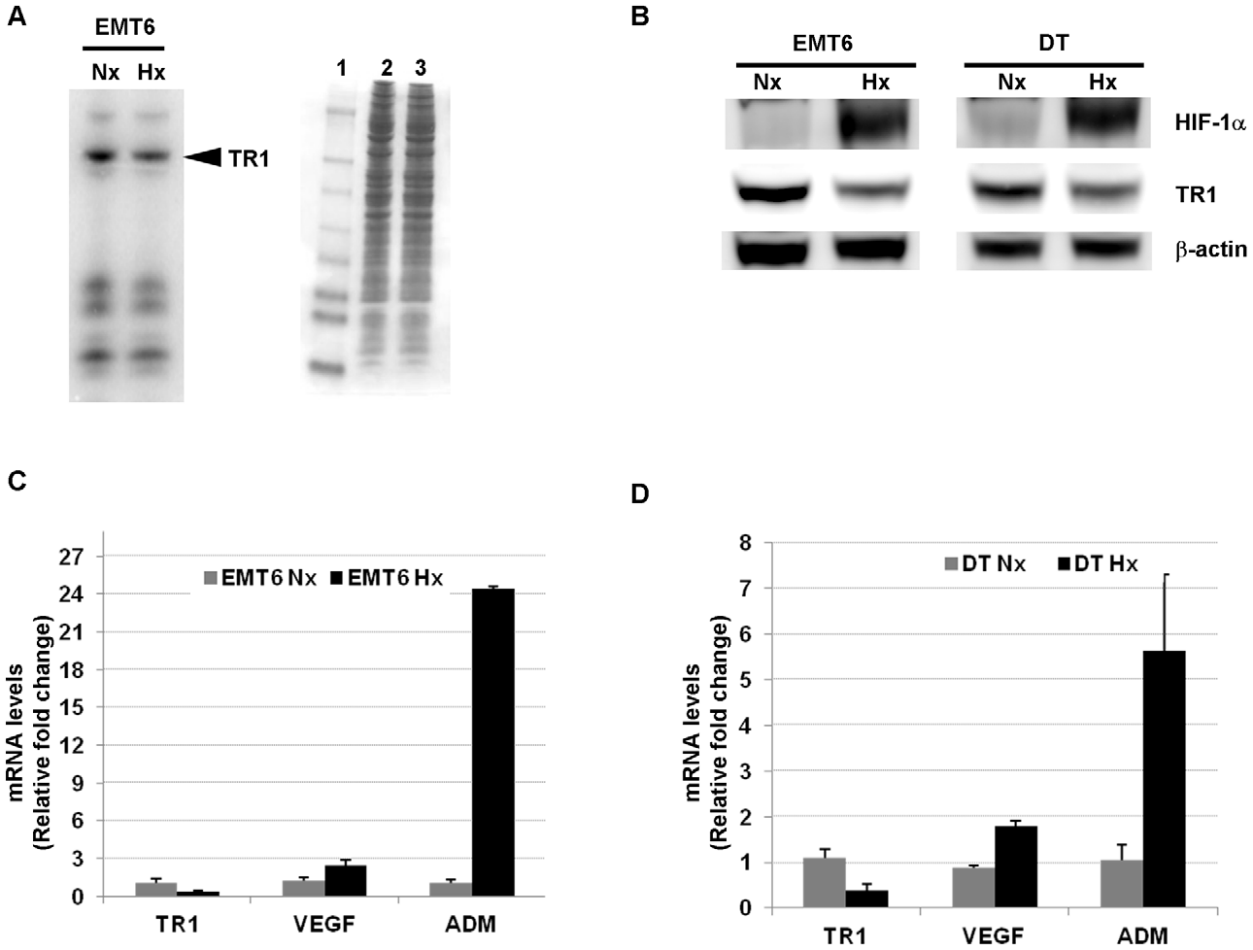
Effect of hypoxic conditions on TR1 mRNA and protein levels. (A) EMT6 cells were labeled with ^75^Se and incubated under normoxic (Nx) or hypoxic (Hx) conditions for 12 h. Cells were lysed and selenoprotein levels analyzed by autoradiography (left panel). Protein standards are shown in lane 1 and protein extracts of EMT6 cells cultured in (lane 2) normoxic or (lane3) hypoxic conditions (right panel). (B) TR1 protein levels were analyzed by western blotting in EMT6 and DT cell lines grown under normoxic (Nx) or hypoxic (Hx) conditions for 12 h. (C) EMT6 and (D) DT cell lines were grown under normoxic (Nx) or hypoxic (Hx) conditions and the mRNA levels of TR1, VEGF and ADM determined by quantitative RT-PCR and normalized to the content of β-actin mRNA in the same sample. The data represent the fold induction of each mRNA over the mean from the normoxic values. The mean from 3 different experiments ± SE is shown.

EMT6 cells were metabolically labeled with ^75^Se and then incubated under normoxic (21% O_2_) or hypoxic (1% O_2_) conditions for 12 h (Fig. 1A). Hypoxia treatment appeared to cause a decrease in TR1 levels, whereas the expression of other selenoproteins was not significantly affected. The reduction in TR1 protein levels by the hypoxic condition was confirmed by western blotting using specific antibodies against TR1 in both EMT6 and DT cell lines (Fig. 1B).

To assess whether hypoxia treatment affected TR1 gene expression levels, we determined TR1 mRNA levels under normoxic or hypoxic conditions. Quantitative RT-PCR showed a significant decrease of TR1 mRNA levels in the cells grown under hypoxic conditions compared to the levels found in the cells grown under normoxic conditions (Fig. 1C and 1D). To monitor the hypoxic conditions we measured the mRNA levels of vascular endothelial growth factor (VEGF) and adrenomedullin (ADM), as both genes are well known to be induced under hypoxic conditions [19]–[21]. As expected, EMT6 and DT cells exposed to hypoxia showed a significant increase in both VEGF and ADM mRNA levels (Fig. 1C and 1D).

### HIF is neither sufficient nor necessary for hypoxia-induced TR1 down-regulation

**Figure 2:**
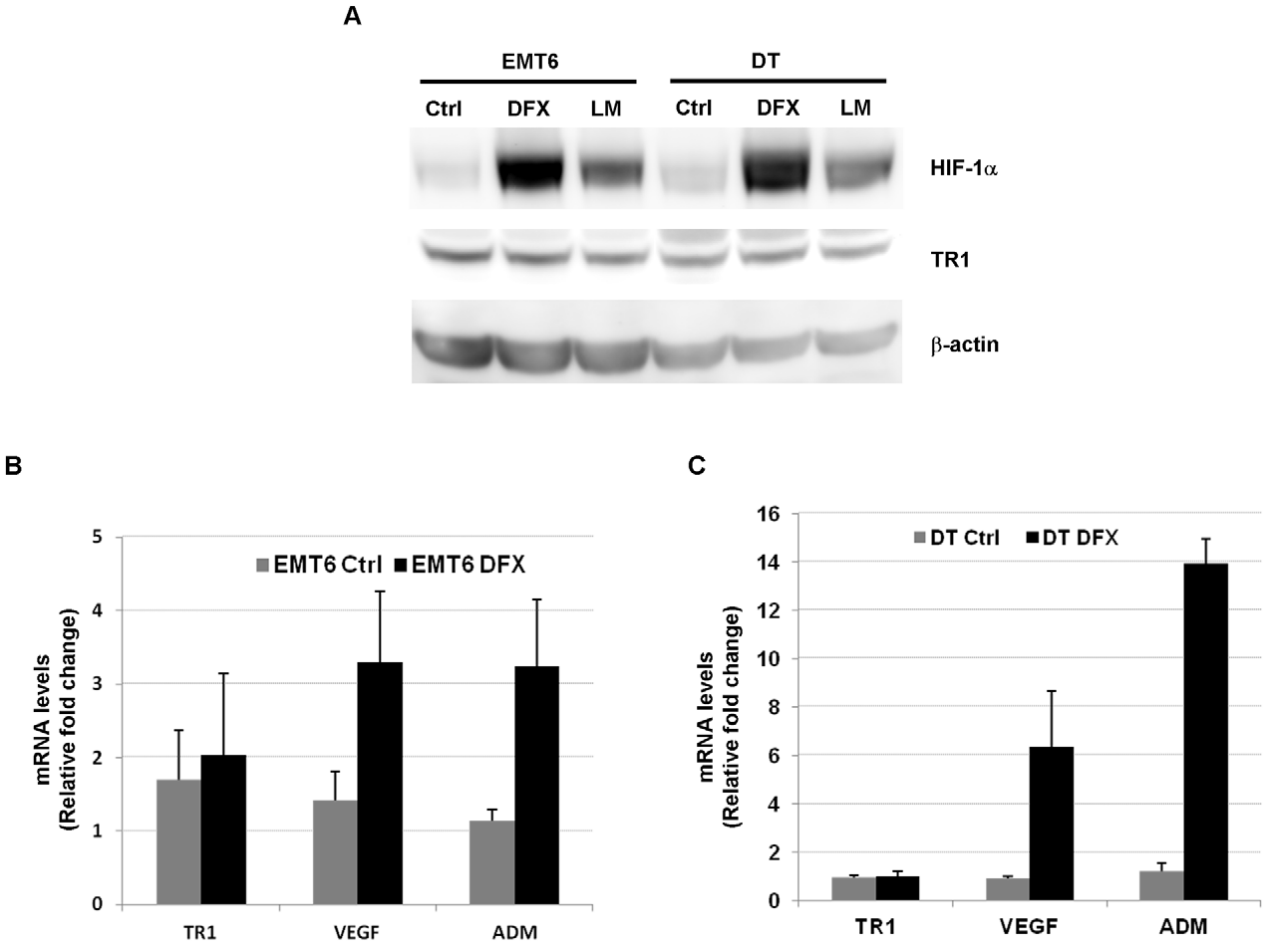
Effect of HIF-1α stabilization by EGLN inhibitors on TR1 expression. (A) EMT6 and DT cells were treated with 150 µM of deferoxamine (DFX), 100 µM of L-mimosine (LM) or left untreated (Ctrl) for 12 h and the levels of HIF-1α and TR1 protein detected by western blotting. The mRNA levels of TR1, VEGF and ADM from (B) EMT6 cells and (C) DT cells, treated with deferoxamine (DFX) or left untreated (Ctrl) for 8 h, were measured by quantitative RT-PCR. The data represent the fold-change relative to untreated cells after normalization with the β-actin content. The mean and ± SE of 3 independent experiments is shown.

Due to the major role of HIF in the regulation of hypoxia-induced changes in gene expression, we investigated whether the reduction of TR1 expression under hypoxia was HIF-dependent. The inhibition of EGLNs leads to HIF stabilization and induction of HIF-target genes [22]. We therefore assessed whether the use of EGLN inhibitors would have the same effect on TR1 levels as hypoxia treatment. DT and EMT6 were treated with the iron chelator deferoxamine or the 2-oxoglutarate analog L-mimosine. As shown in Fig. 2A, deferoxamine and L-mimosine treatment resulted in HIF-1α stabilization in both, DT and EMT6 cell lines. This stabilization of HIF-1α correlated with an increase in the expression of HIF-target genes, VEGF and ADM, in DT (Fig. 2B) and EMT6 cells (Fig. 2C). However, the treatment with deferoxamine or L-mimosine in DT and EMT6 did not affect significantly TR1 mRNA (Fig. 2B and 2C) or protein levels (Fig. 2A), indicating that HIF stabilization/activity is not sufficient for TR1 down-regulation.

**Figure 3:**
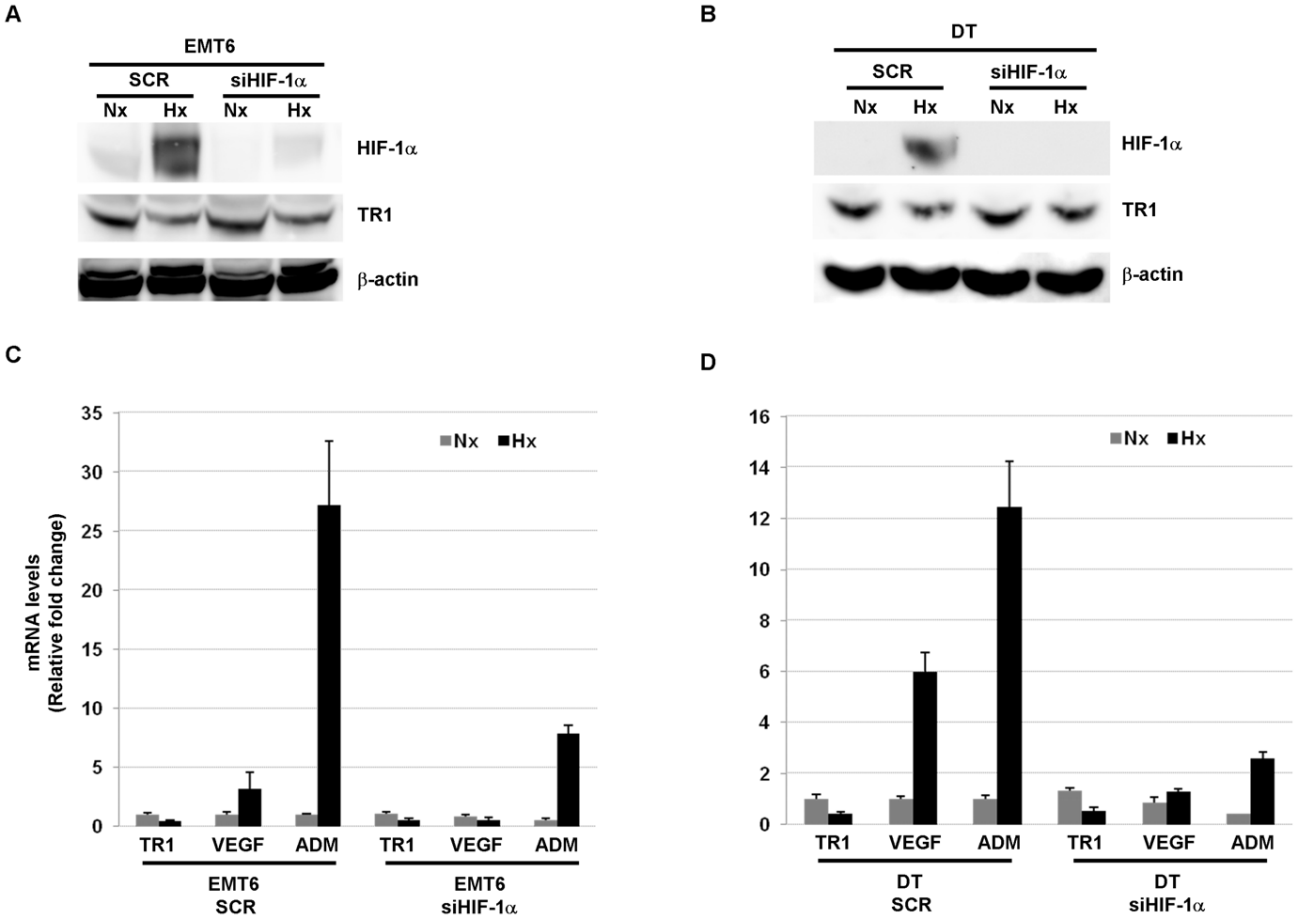
Hypoxic regulation of TR1 expression in HIF-1α deficient cells. TR1 and HIF-1α protein levels, from (A) EMT6 cells and (B) DT cells infected with retrovirus encoding a scramble sequence (SCR) or a HIF-1α shRNA (si HIF-1α) and cultured under normoxic (Nx) or hypoxic (Hx) conditions for 12 h, were determined by western blotting. (C) EMT6 cells or (D) DT cells infected with the indicated retroviruses were cultivated for 12 h under normoxic (Nx) or hypoxic conditions (Hx) and TR1, VEGF and ADM mRNA levels measured by quantitative RT-PCR. The fold-change relative to the normoxic values and normalized with the β-actin is shown. Data represent the mean and ± SE of 3 independent experiments.

We then examined whether HIF-1α was necessary for the hypoxia-induced decrease of TR1 levels. To this end, EMT6 and DT cells were infected with retroviruses encoding siHIF-1α or a scrambled sequence (SCR). HIF-1α knockdown cells exposed to hypoxia showed a decrease in HIF-1α protein levels (Fig. 3A and 3B) compared to respective control cells. This reduction in HIF-1α protein correlated with a reduced hypoxic-induction of HIF-target genes, VEGF and ADM, in HIF knockdown cells compared to the cells infected with the retrovirus encoding the scramble sequence (Fig. 3C and 3D). However, no significant effect was observed in the hypoxia-induced reduction of TR1 protein (Fig. 3A and 3B) and mRNA levels (Fig. 3C and 3D).

### TR1 activity is reduced in cells exposed to hypoxia

**Figure 4:**
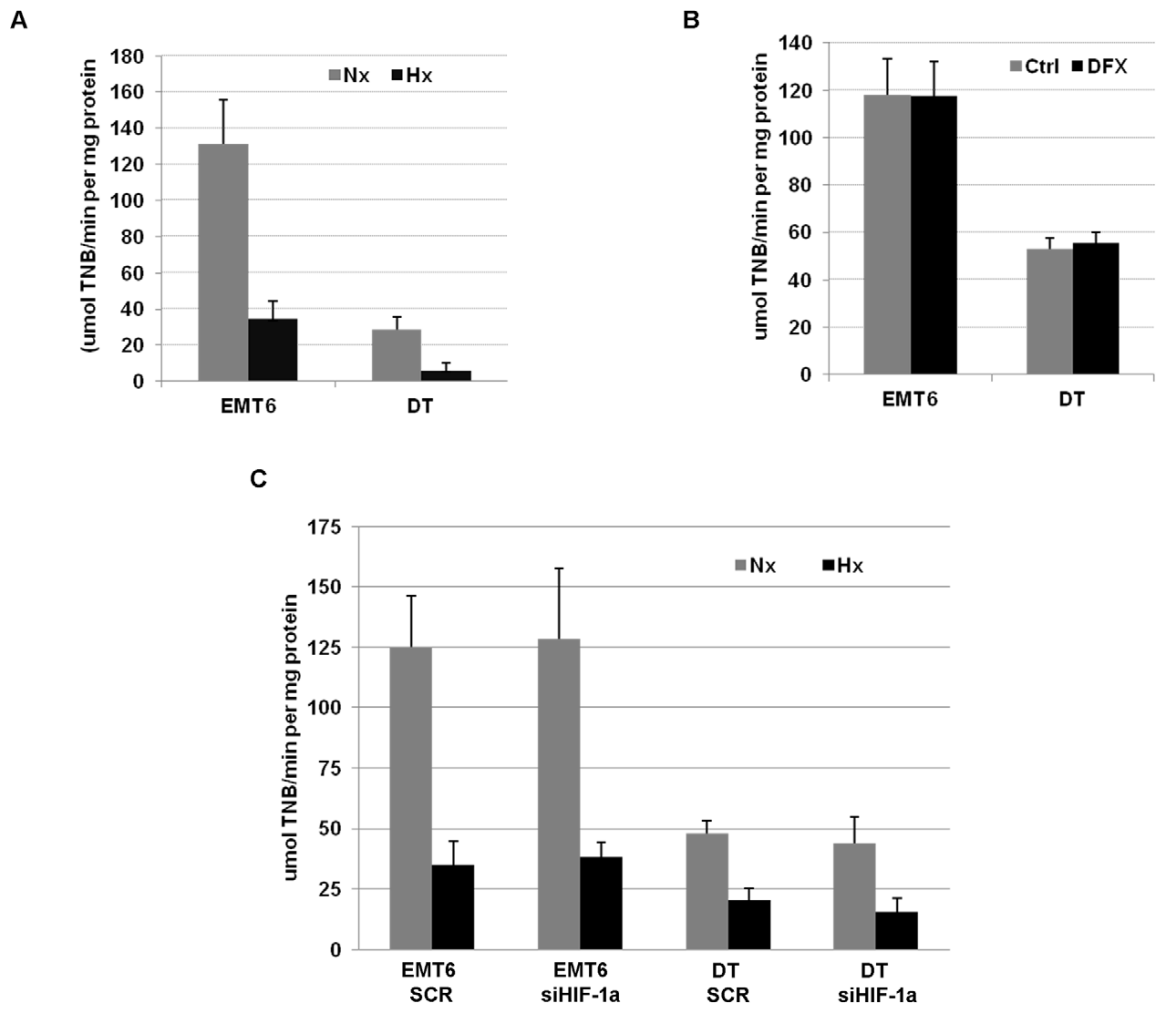
Effect of hypoxic conditions, EGLN inhibitors or HIF-1α deficiency on TR1 activity. (A) TR1 activity was measured as indicated in Methods in EMT6 and DT cells grown under normoxic (Nx) or hypoxic (Hx) conditions for 12 h. (B) EMT6 and DT cells were treated with 150 µM deferoxamine (DFX) or left untreated (Ctrl) for 12 h and TR1 activity measured. (C) EMT6 and DT cells infected with retrovirus encoding a scramble sequence (SCR) or a HIF-1α shRNA (si HIF-1α) were cultured under normoxic (Nx) or hypoxic (Hx) conditions for 12 h and TR1 activity measured. Data represent the means of 3 independent experiments ± SE.

To assess whether the reduction in TR1 levels resulted in a reduction of the activity of this enzyme, we determined TR1 activity in EMT6 and DT cells cultured under hypoxic conditions. As shown in Fig. 4A, hypoxia treatment significantly reduced TR1 activity in both EMT6 and DT cells. On the contrary, the use of the EGLNs inhibitor, deferoxamine, did not affect TR1 activity (Fig. 4B). Furthermore, EMT6 and DT HIF-1α knockdown cells exposed to hypoxic conditions showed a decrease in TR1 activity similar to the decrease observed in the hypoxic treated control cells (Fig. 4C). These results show that the reduction in TR1 mRNA and protein levels under hypoxic conditions also resulted in a decrease in TR1 activity, and that this reduction in TR1 activity, as the decrease in TR1 mRNA and protein levels, is HIF-1α independent.

### TR1 deficiency increases the hypoxia-induced generation of ROS

**Figure 5:**
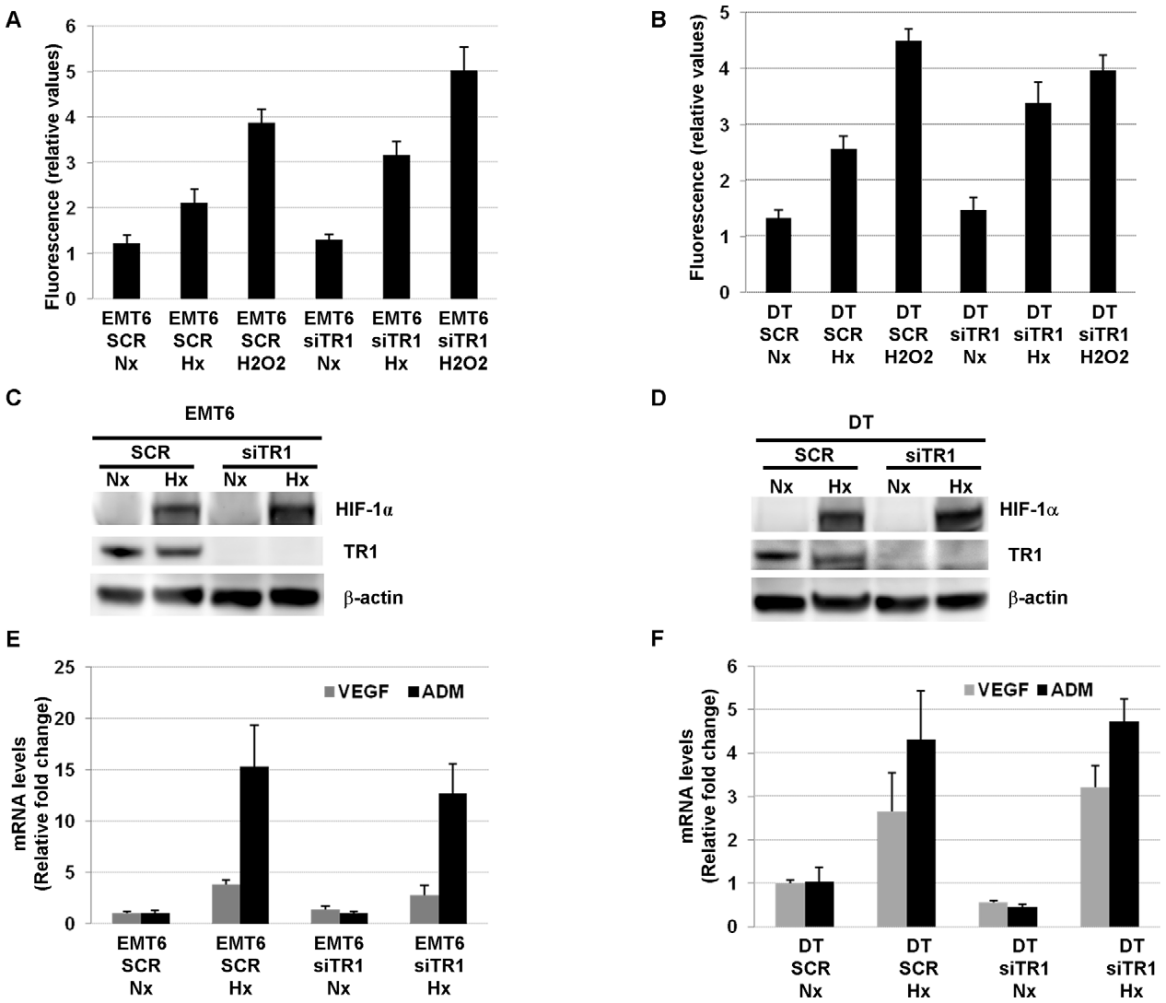
Effect of TR1 deficiency on ROS levels and HIF activity in cells cultured under hypoxic conditions. (A) EMT6 and (B) DT cells infected with retroviruses encoding a scramble sequence (SCR) or a TR1 shRNA (siTR1) were grown under normoxic (Nx) or hypoxic (Hx) conditions for 12 h and ROS levels determined as described in Methods. As a positive control for ROS generation, cells were treated with 100 µM of H_2_O_2_ where indicated. Data represent the fluorescence value of each sample after subtracting the fluorescence value from unstained control cells. The mean ± SE of 3–4 independent experiments is shown. HIF-1α, TR1 and β-actin protein levels were determined by western blotting in (C) EMT6 and (D) DT cells infected with retroviruses encoding a scramble sequence (SCR) or a TR1 shRNA (siTR1) and cultured under normoxic (Nx) or hypoxic (Hx) conditions for 12 h. mRNA levels of the HIF target genes, VEGF and ADM, were analyzed by quantitative RT-PCR in (E) EMT6 or (F) DT cell lines infected with retroviruses encoding a scramble sequence (SCR) or a TR1 shRNA (siTR1) and cultured under normoxic (Nx) or hypoxic (Hx) conditions for 12 h. Data show the means of 3 independent experiments ± SE.

In order to study the importance of hypoxia-induced down-regulation of TR1 in the increase of ROS levels observed under low oxygen levels conditions, we generated EMT6 and DT cells in which TR1 expression was knocked down reducing TR1 mRNA levels more than 80% compared to control cells (Fig. S1). Both cell lines were cultured under normoxic or hypoxic conditions and the highly sensitive H2DCFDA probe was used for measuring intracellular ROS levels [23]–[24]. As shown in Fig. 5A and 5B, although hypoxia significantly increased ROS levels in both control and TR1 knockdown cells, the increase in ROS levels in TR1 knockdown cells cultured under hypoxic conditions was significantly higher than in the control cells. The knockdown of TR1, however, did not increase significantly the ROS levels under normoxic conditions (Fig. 5A and 5B).

ROS generated under hypoxic conditions have been shown to be required for the inhibition of the activity of the EGLN enzymes responsible for hypoxic HIF stabilization. Considering the increase that we observed in the hypoxic ROS levels in cells in which TR1 was knocked down compared to control cells, we assessed whether HIF stabilization or activity could be increased under these conditions. However, we were unable to find differences in HIF protein levels (Fig. 5C and 5D) or in the induction of the HIF target genes, VEGF and ADM, in the TR1 knockdown cells compared to the control cells (Fig. 5E and 5F).

### TR1 overexpression inhibits the increase in ROS induced by hypoxia

**Figure 6:**
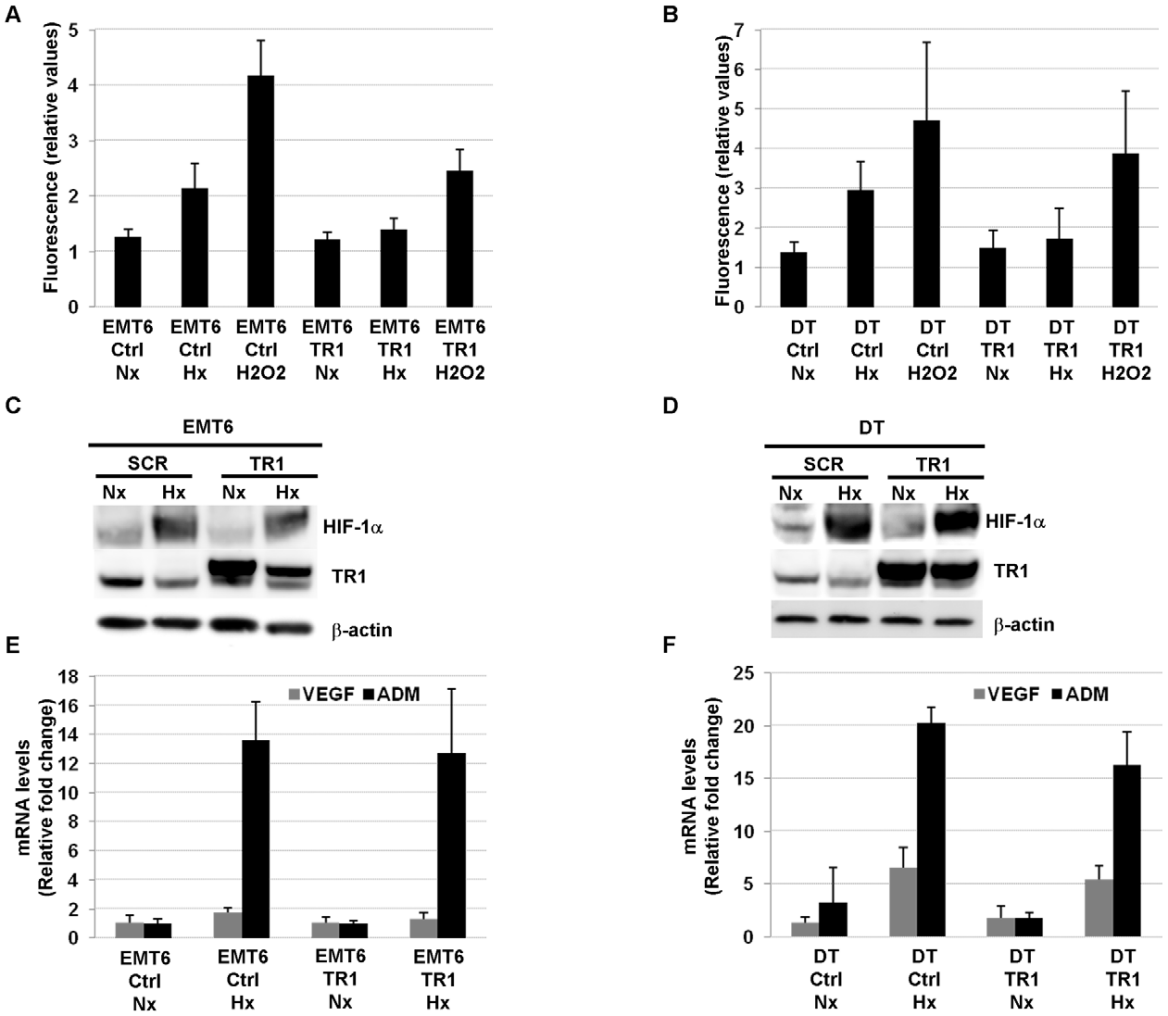
ROS levels and HIF stabilization and activity in hypoxic TR1 over-expressing cells. ROS levels were determined in (A) EMT6 and (B) DT cells infected with retroviruses encoding the TR1 gene (TR1) or empty retroviruses (Ctrl) and cultured under normoxic (Nx) or hypoxic (Hx) conditions for 12 h. Data represent the fluorescence value of each sample after subtracting the fluorescence value from unstained control cells. The mean ± SE of 4–7 independent experiments is shown. Protein levels of HIF-1α and TR1 were determined by western blotting in (C) EMT6 and (D) DT cells infected with empty retroviruses (Ctrl) or retroviruses encoding the TR1 gene (TR1) and cultured for 12 h under normoxic (Nx) or hypoxic (Hx) conditions. VEGF and ADM mRNA levels were analyzed by quantitative RT-PCR in EMT6 (E) or DT (F) cell lines with retroviruses encoding the TR1 gene (TR1) or with empty retroviruses (Ctrl) and cultured under normoxic (Nx) or hypoxic (Hx) conditions for 12 h. Data show the means of 3 independent experiments ± SE.

Due to the effect on the ROS generation under hypoxic conditions observed in TR1 deficient cells, we examined whether the over-expression of TR1 could affect the hypoxia-induced generation of ROS. We therefore generated EMT6 and DT TR1 over-expressing cells. TR1 mRNA and protein levels were highly elevated in over-expressing cells compared to control cells (Fig. S2). We then exposed control and TR1 over-expressing cells to hypoxic or normoxic conditions and total ROS production was analyzed as described in Methods. As a positive control for ROS generation cells were treated with H_2_O_2_ for 10 minutes. This treatment resulted in a significant increase in the ROS levels in both control and TR1 over-expressing cell lines (Fig. 6A and 6B). However, hypoxic conditions significantly elevated ROS only in the control cells, but not in the TR1 over-expressing cells (Fig. 6A and 6B).

Other studies have shown that ROS generated under hypoxic conditions at the mitochondrial level are essential for HIF stabilization. Since we did not observe a significant increase in ROS levels in TR1 over-expressing cells under hypoxic conditions compared to normoxic conditions, we examined how TR1 over-expression affected HIF stabilization or activity. DT and EMT6 TR1 over-expressing cells were cultured under normoxic or hypoxic conditions and HIF-1α protein levels analyzed by western blotting. No significant changes in HIF-1α protein levels were found in TR1 over-expressing DT or EMT6 cells compared to control cells (Fig. 6C and 6D). Furthermore, the analysis of the mRNA of the HIF target genes VEGF and ADM demonstrated that the activity of HIF was not affected by blocking the production of ROS under hypoxic conditions by over-expressing TR1 (Fig. 6E and 6F).

## Discussion

To determine whether hypoxia affects selenoprotein expression, we cultured ^75^Se-labeled EMT6 cells under hypoxic conditions and found that TR1 levels were significantly reduced compared to the TR1 levels of EMT6 cells cultured under normoxic conditions. This reduction in TR1 protein levels was also found by western blotting using a specific antibody against TR1. Furthermore, quantitative PCR demonstrated a down-regulation of TR1 mRNA levels, suggesting that the decrease in TR1 levels is achieved through transcriptional regulation.

HIF is the major transcription factor implicated in gene expression changes induced by hypoxia. Although most of its target genes are up-regulated after HIF stabilization and activation [25], there are also genes that have been shown to be down-regulated in a HIF-dependent manner under hypoxic conditions [26], [27]. In our study we showed, by inhibiting the activity of the EGLNs, that HIF stabilization was not sufficient to reduce TR1 levels. Moreover, the use of a retrovirus to knockdown the expression of HIF-1α did not have any effect on the hypoxia-induced decrease of TR1 levels. Therefore, HIF is neither sufficient nor required for TR1 regulation upon hypoxic conditions.

In addition, we found a decrease in TR1 activity in cells cultured under hypoxic conditions that was also independent of HIF stabilization or activity. Interestingly, TR1 activity was decreased to a greater extent than its mRNA and protein levels, suggesting that the hypoxic effects on TR1 could involve mechanisms other than a change in expression levels; e.g., translational or post-translational modification of TR1 that affects its activity. In addition, other components involved in the thioredoxin reductase reaction could be affected by hypoxic conditions. Further study is required to elucidate the precise mechanism(s) responsible for this anomaly.

To our knowledge, this is the first report showing a down-regulation of TR1 protein and mRNA by hypoxic conditions. This result suggests that the increase in ROS levels observed under hypoxic conditions could be due not only to an increase in ROS generation, but also to a decrease in the activity in one of the major cellular antioxidant systems, e.g. the thioredoxin/thioredoxin reductase system [28]. Although ETC is frequently cited as the most important source of ROS (see [29] and references therein), the NADPH oxidase system can also produce intracellular ROS in some cell types and several studies have found a reduction in the specific ROS generated by this system after hypoxic treatment [30]–[32]. Under such conditions, an increase in ROS could be explained by a reduction in the activity of the systems required to reduce intracellular ROS levels. In agreement with this proposal, we found that TR1 knockdown elevated the ROS levels induced by hypoxia whereas the over-expression of TR1 was able to avoid ROS accumulation under hypoxic conditions. However, this result does not exclude the possibility of an increase in the generation of ROS in the mitochondrial in response to hypoxic conditions.

It has been reported that the increase in ROS levels observed under hypoxic conditions could be crucial for cellular oxygen sensing and the activation of the hypoxic adaptive response [33], [34]. These earlier studies maintained that ROS generated in hypoxia are required for the complete inhibition of the activity of the EGLNs. However the Km values for O_2_ of the three EGLNs have been shown to be close to the atmospheric oxygen concentration, and thus even small decreases in O_2_ levels are likely to influence their activities [35]. In contrast to these studies, we found that a larger increase in ROS levels under hypoxic conditions, as a result of down-regulating TR1 expression, did not have an effect on HIF-1α stabilization or activity. Furthermore, the over-expression of TR1, that was able to block the increase in ROS levels under hypoxia, did not affect HIF-1α protein stabilization or activity. These results suggest that ROS generated under hypoxic conditions are not required for the inhibition of the activity of EGLNs.

Thioredoxin/thioredoxin reductase system also plays a critical role in the generation of deoxyribonucleotides, needed for DNA synthesis, and is essential for cell proliferation [36]. A down-regulation of TR1 under hypoxic conditions could be a possible mechanism to induce cell cycle arrest. In fact, it has been described previously that hypoxic conditions induce cell cycle arrest [37], although there are studies showing that this cell cycle arrest could be HIF-dependent [38].

On the other hand, the reduction in TR1 activity induced by hypoxia, and the subsequent increase on intracellular H_2_O_2_, could act as a positive feedback for some of the signaling pathways activated under hypoxic conditions. In agreement with this hypothesis, H_2_O_2_ has been shown to play an important role in angiogenesis. Thus, H_2_O_2_ stimulates angiogenic responses in cultured endothelial cells and smooth muscle cells and induces the production of VEGF protein in different cell types [39]. Furthermore, an important role of peroxide signaling is the potentiation of receptor tyrosine kinase signaling, including VEGF receptor 2, by oxidation of protein tyrosine phosphatases (PTP) that negatively regulate their signaling [40].

Finally, the thioredoxin/thioredoxin reductase 1 system has an important role in transcriptional regulation. Some cysteine residues of several transcriptions factors, such as NF-κB, glucocorticoid receptor, AP1 or p53 [36], are reduced by thioredoxin, and the DNA binding, of these factors, usually require these cysteine residues to be in a reduced state. Therefore, the decrease in TR1 activity under hypoxic conditions that we have observed could be a mechanism to regulate the transcriptional activity of these transcription factors. In the case of p53, a reduction in its transcriptional activity could be critical for tumor cells to avoid apoptosis and progress under hypoxic conditions.

## Supporting Information

Figure S1 **Knockdown of TR1 in EMT6 and DT cells.** EMT6 and DT cells were infected with retroviruses encoding a TR1 shRNA (siTR1) or a scramble sequence as a control. RNA was collected from control and knockdown cells and TR1 mRNA levels determined by quantitative PCR and normalized by the content of β-actin. A value of 1 (solid line) was assigned to the normalized TR1 level of control cells. The normalized levels of TR1 in knockdown cells samples are represented as fold over control samples. Data show the results obtained in three independent experiments and their average values.
(TIF)

Figure S2 **Over-expression of TR1 in EMT6 and DT cells.** EMT6 and DT cells were infected with empty retroviruses (Ctrl) or with retroviruses encoding the TR1 gene (TR1). (A) TR1 mRNA levels were measured by quantitative RT-PCR and normalized by the content of β-actin. Data show the average fold-change over control of three independent experiments ± SE. (B) TR1 and β-actin protein levels were determined by western blotting in EMT6 or DT TR1 over-expressing cells.
(TIF)
