# Supplementary figures and images for: HIF-Independent Regulation of Thioredoxin Reductase 1 Contributes to the High Levels of Reactive Oxygen Species Induced by Hypoxia
Source: PLoS One. 2012 Feb 13;7(2):e30470. doi: 10.1371/journal.pone.0030470 (PMC3278416; doi:10.1371/journal.pone.0030470)

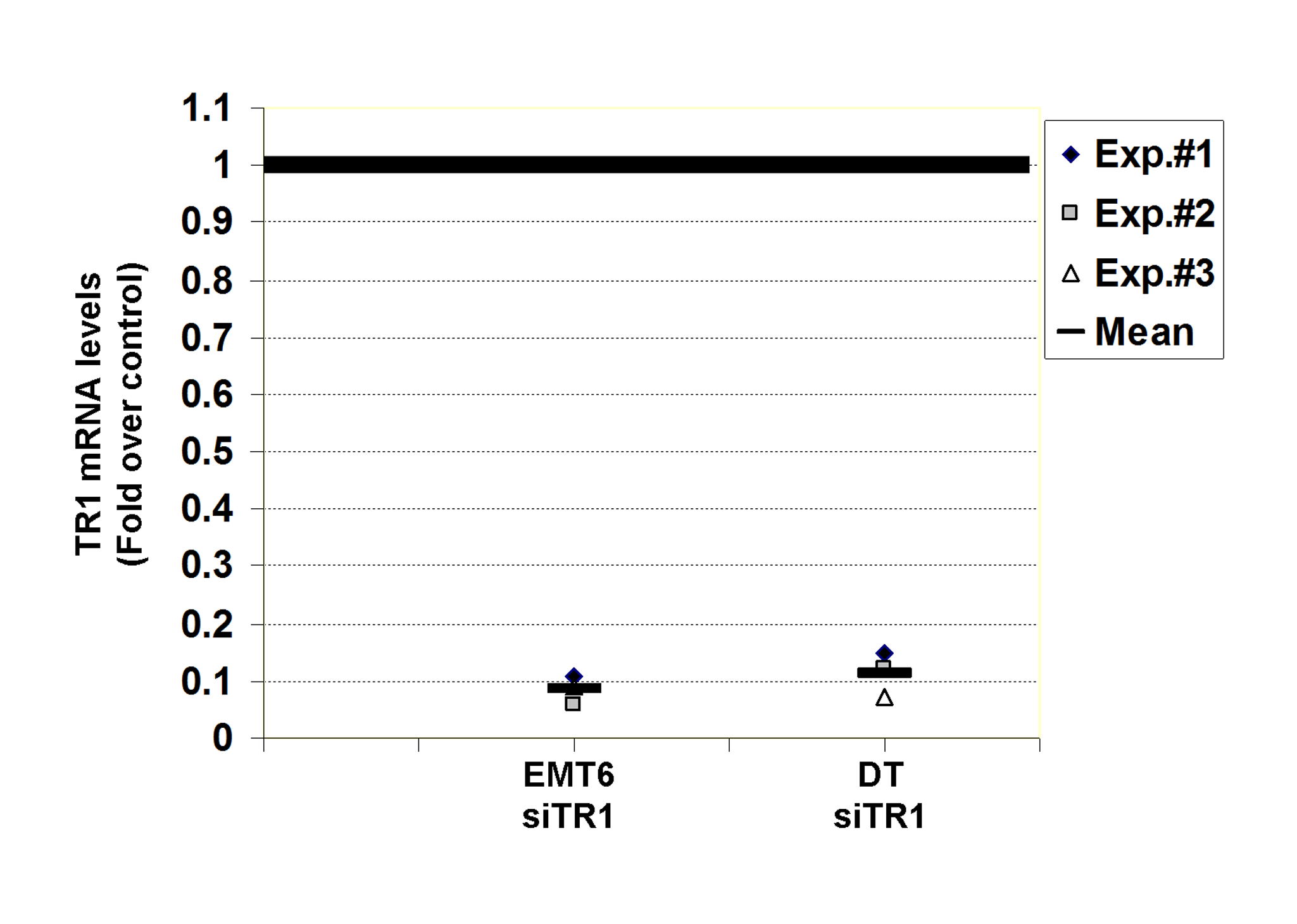

Supplement: Figure S1 — Knockdown of TR1 in EMT6 and DT cells. EMT6 and DT cells were infected with retroviruses encoding a TR1 shRNA (siTR1) or a scramble sequence as a control. RNA was collected from control and knockdown cells and TR1 mRNA levels determined by quantitative PCR and normalized by the content of β-actin. A value of 1 (solid line) was assigned to the normalized TR1 level of control cells. The normalized levels of TR1 in knockdown cells samples are represented as fold over control samples. Data show the results obtained in three independent experiments and their average values. (TIF) [file pone.0030470.s001.tif]

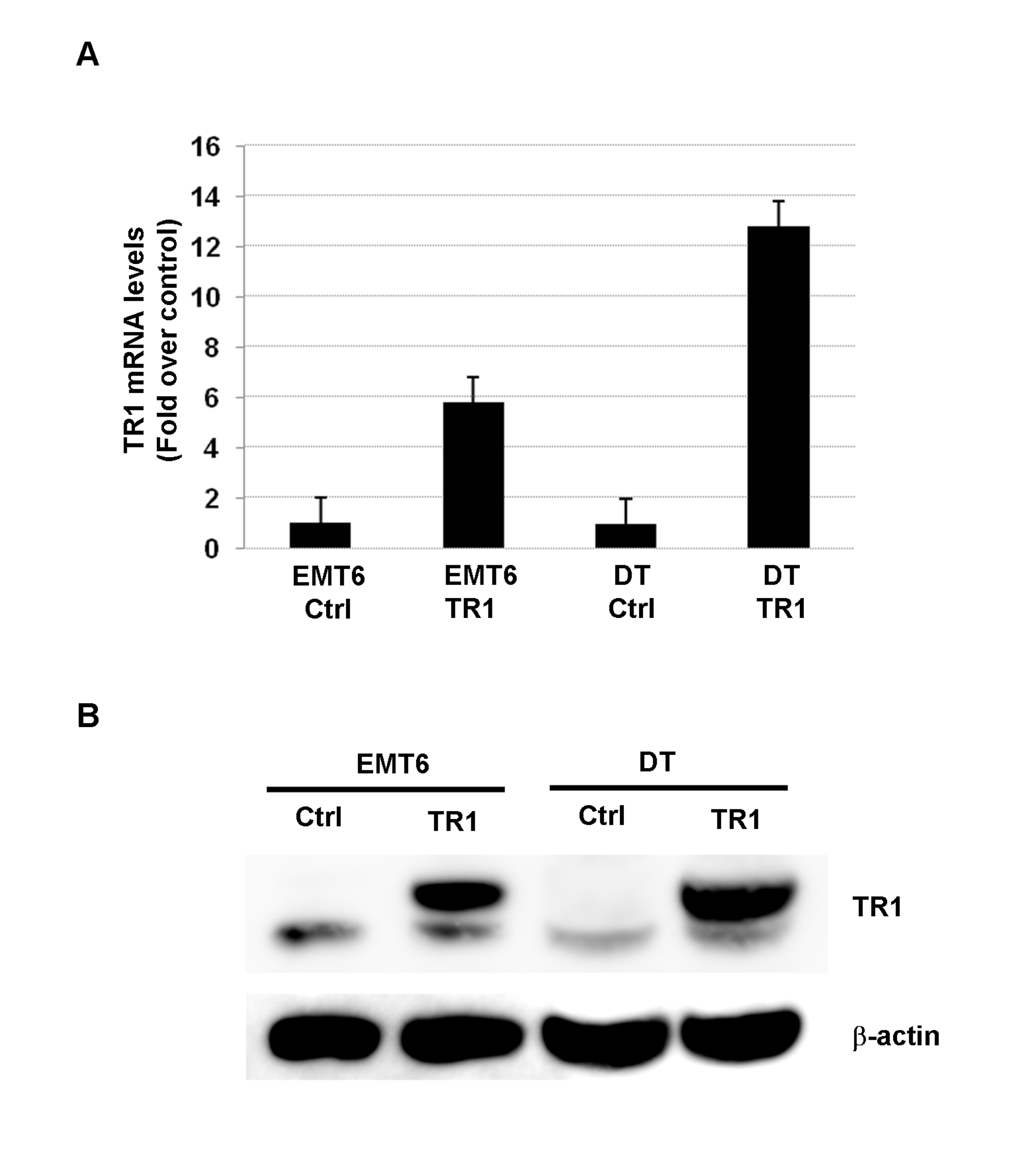

Supplement: Figure S2 — Over-expression of TR1 in EMT6 and DT cells. EMT6 and DT cells were infected with empty retroviruses (Ctrl) or with retroviruses encoding the TR1 gene (TR1). (A) TR1 mRNA levels were measured by quantitative RT-PCR and normalized by the content of β-actin. Data show the average fold-change over control of three independent experiments ± SE. (B) TR1 and β-actin protein levels were determined by western blotting in EMT6 or DT TR1 over-expressing cells. (TIF) [file pone.0030470.s002.tif]
